# Supplementary material for: Mineralogical controls of the oceanic nickel cycle
Source: Nat Commun. 2025 Aug 14;16:7552. doi: 10.1038/s41467-025-62842-3 (PMC12354895; doi:10.1038/s41467-025-62842-3)
Supplement: Supplementary file 1 — Supplementary Information [file 41467_2025_62842_MOESM1_ESM.pdf]

SUPPLEMENTARY INFORMATION 1

Supplementary Table 1. Selected elemental concentrations and Ni isotope data of bulk Mn oxide sediments used in this study, from the Pacific Ocean, the MANOP sites and the USGS reference Mn nodules, measured previously by Fleischmann et al.[1] and Little et al.[2] X-ray diffraction data is available for the MANOP samples in Little et al.[2] and for the USGS nodules in Knaack et al.[3]

Sample Sites	Sediment Depth	Location	Water Depth	Sample Type	Al (%)	Fe (%)	Mn (%)	Ni (ppm)	δ^{60}Ni (‰)	2σ
**Pacific Ocean, open ocean sediments**										
DWHG-49A	2-4 cm	42° 02' S, 98° 01' W	4350 m	Deep-sea sediments	1.4	6.5	2.1	509	0.42	0.07
DWBG-30A	2-4 cm	19° 50' S, 148° 39' W	4620 m	Deep-sea sediments	5.6	7.4	1.0	250	0.89	0.05
CK-4	10-15 cm	42° 30' N, 162° 08' W	5350 m	Deep-sea sediments	9.8	5.0	0.2	95	0.40	0.05
DWBG-2C	2-4 cm	21° 27' N, 126° 43' W	4370 m	Deep-sea sediments	9.8	5.5	0.6	223	0.92	0.07
VM18-258	8-10 cm	11° 52' S, 165° 45' W	5528 m	Deep-sea sediments	6.8	8.1	1.4	207	0.81	0.06
VM21-71	8-10 cm	27° 54' N, 162° 31' E	5860 m	Deep-sea sediments	9.0	5.3	0.5	130	0.46	0.07
**Pacific Ocean, continental margin sediments, MANOP sites**										
Manop-H	4 -5 cm	6° 30' N, 92° 48' W	~ 3600 m	Margin sediments	5.3	4.1	4.8	872	-0.17	0.04
	9-11 cm				5.3	4.2	5.3	785	-0.21	0.04
	13-15 cm				4.8	3.8	2.1	379	-0.27	0.05
Manop-M	0.5-1 cm	8° 48' N, 104° 00' W	~ 3100 m	Margin sediments	4.2	5.5	1.4	286	-0.42	0.04
	3 -5 cm				4.4	5.6	1.5	287	-0.45	0.04
	9-11 cm				4.2	5.4	0.9	228	-0.63	0.04
**USGS Mn Nodules**										
USGS NodA1	Surface	31°02'N, 78°22' W	788 m	Mn Nodules	1.63	9.8	19.8	5732	1.06	0.07
USGS NodP1	Surface	14°50' N, 124°28' W	4340 m	Mn Nodules	1.36	4.9	30.1	11559	0.34	0.05

Supplementary Table 2 Fraction of Ni adsorbed vs $\Delta^{60}Ni_{mineral-aqueous}$ (‰) for synthetic samples and natural sediments from this study.

**Sample Grouping**	**Sample ID**	**Fraction of Ni adsorbed to the mineral**	**Error**	**$\Delta^{60/58}Ni_{min-aq}$ (mineral - aqueous)**	**Error**
Pacific Ocean Sediments	DWBG 2C (2-4) 1	0.29	0.20	-0.41	0.21
Pacific Ocean Sediments	DWBG 49A (2-4) 1	0.14	0.20	-0.91	0.21
Pacific Ocean Sediments	DWBG 30A(2-4) 1	0.27	0.20	-0.44	0.21
Pacific Ocean Sediments	VH18-256 (8-10) 1	0.24	0.20	-0.52	0.21
Pacific Ocean Sediments	VH21-71	0.37	0.20	-0.87	0.21
Pacific Ocean Sediments	CK4 1	0.29	0.20	-0.93	0.21

Site H	VULCAN 37BC MP 8951	0.46	0.20	-1.50	0.21
Site H	VULCAN 37BC MP 5308	0.40	0.20	-1.54	0.21
Site H	VULCAN 37BC MP 5310	0.59	0.20	-1.60	0.21
Site M	PLUTO 20BC MP 8966	0.53	0.20	-1.75	0.21
Site M	PLUTO 20BC MP 8968	0.54	0.20	-1.78	0.21
Site M	PLUTO 20BC MP 8971	0.41	0.20	-1.96	0.21
Nod P-1		0.38	0.20	-0.99	0.21
Nod A-1		0.21	0.20	-0.27	0.21
Synthetic Samples	Ni-Bn 1	0.91	0.06	-1.80	0.16
Synthetic Samples	Ni-Bn 2	0.57	0.01	-1.13	0.16
Synthetic Samples	Ni-Bn 3	0.38	0.06	-1.50	0.16

10

SUPPLEMENTARY INFORMATION 2

Supplementary Figure S1. Ni K-edge EXAFS and Fourier transforms of the EXAFS for our three Ni-birnessite synthetic test samples and two natural sediments (USGS Mn nodules NOD P1 and NOD A1). Ni-Bn 1, Ni-Bn 2 and Ni-Bn 3 are the three Ni-birnessite synthetic test samples, NOD P1 and NOD A1 are the two natural sediments. Solids lines are data, dotted lines are fits.

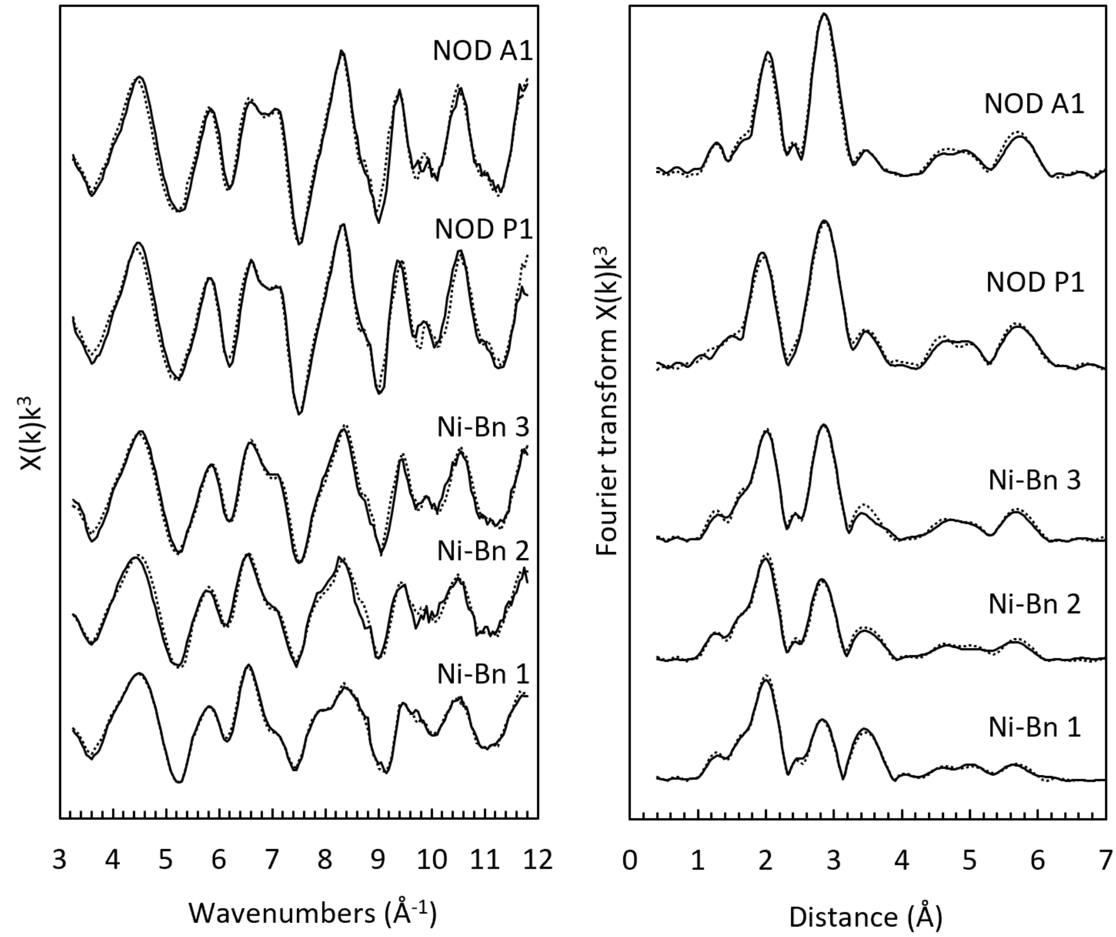

Supplementary Table 3. EXAFS linear combination fits of our three Ni-birnessite synthetic test samples and two natural sediments (USGS Mn nodules NOD P1 and NOD A1). The % adsorbed Ni as determined by wet chemical treatment is given in parentheses after the sample name.

Sample	EF	*N* Ni^{INC}	*N* Ni^{ADS}	R(%)	Reduced Chi^2
Ni-Bn 1 (70±6% adsorbed)	2.56	0.27 ± 0.04	0.73 ± 0.04	21.7	2.2
Ni-Bn 2 (52±4% adsorbed)	2.08	0.40 ± 0.05	0.60 ± 0.05	23.6	2.5
Ni-Bn 3 (39±6% adsorbed)	1.65	0.65 ± 0.04	0.35 ± 0.04	22.4	2.3
NOD P1 (36±2% adsorbed)	2.97	0.80 ± 0.05	0.20 ± 0.05	21.9	2.3
NOD A1 (22±2% adsorbed)	2.37	0.90 ± 0.04	0.10 ± 0.04	22.3	2.3

EF is the correction to the Fermi energy value assigned in the Athena software. *N* Ni^{INC} is the number of Ni atoms (Ni site occupancy) for the model cluster representing Ni structurally incorporated into the phyllomanganate layers. *N* Ni^{ADS} is the number of Ni atoms (Ni site occupancy) for the model cluster representing Ni adsorbed at Mn octahedral vacancies in the phyllomanganate layers.

SUPPLEMENTARY INFORMATION 3

Supplementary Figure S2. Powder XRD pattern for our Ni-birnessite (made with a 35 minute synthesis time, bottom; a 5 minute synthesis time, middle; and a 3 hour synthesis time, top), including characteristic basal reflections [001], [002], [100] and [110], manifesting as four broad characteristic peaks at ~7 Å, ~3.6 Å, ~2.4 Å and ~1.4 Å, respectively, in agreement with previous studies[4–7].

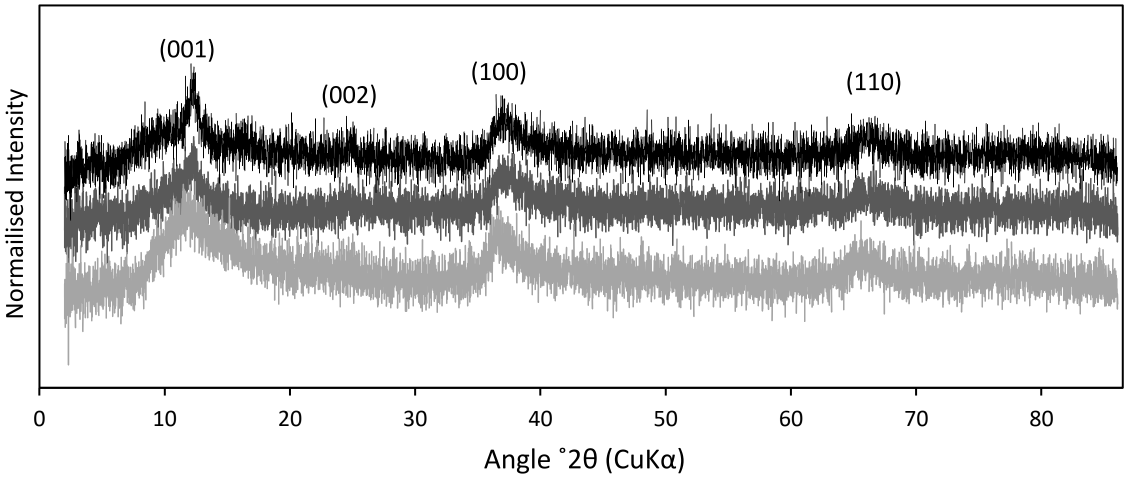

Supplementary Figure S3. Powder XRD patterns for our birnessite to todorokite transformation. Ni-Bn is the Ni-birnessite precursor, 10Å P is the Mg-phyllomanganate intermediate and 3h…4wk refer to the timepoints during the transformation procedure. Characteristic XRD peaks are labelled for birnessite (B), Mg-phyllomanganate intermediate (MgI) and todorokite (T) (based on JCPDS-32-1128 for 10 Å phyllomanganate and JCPDS-38-475 for todorokite). Peaks labelled with a * are from residual $MgCl_2$.

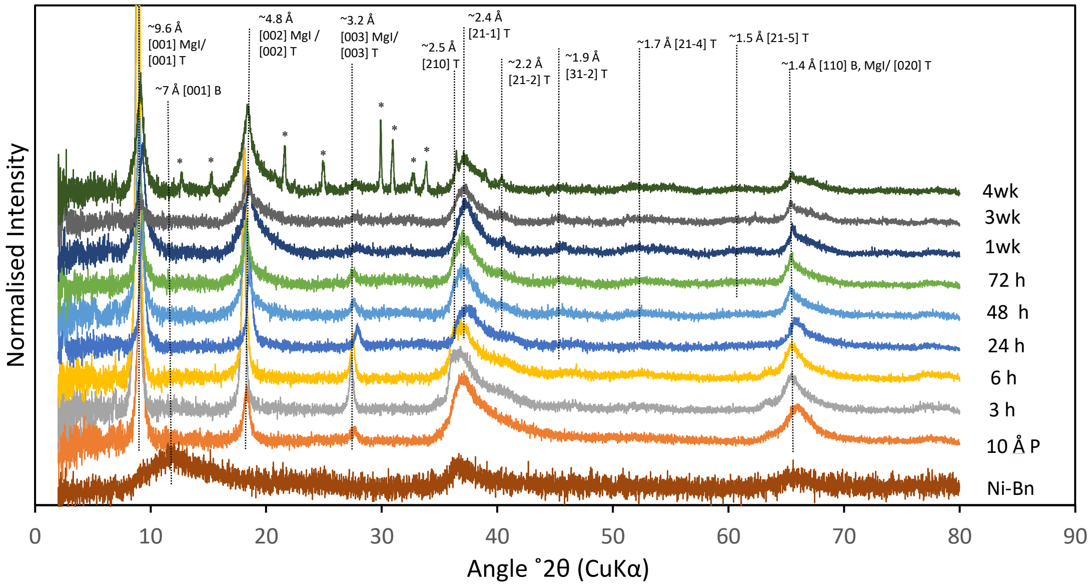

In agreement with previous studies[1–4], our Ni-birnessite precursor shows the four broad characteristic peaks for birnessite at ~7 Å, ~3.6 Å, ~2.4 Å and ~1.4 Å corresponding to the basal reflections [001], [002], [100] and [110]. After suspension of our Ni-birnessite in $MgCl_2$ solution, the interlayer spacing expands from ~7 Å to~ 10 Å. Our Mg-phyllomanganate intermediate thus shows four characteristic peaks for 10 Å phyllomanganate at ~4.8 Å [002], ~3.2 Å [003], ~2.4 Å [100] and ~1.4 Å [110]. Both our Ni-birnessite and Mg-phyllomanganate intermediate have a [110]/[111] reflection ratio of around and the 1.4 Å peak is symmetrical, indicating that both phases have hexagonal symmetry.

In the first 24 h of reflux, there is some evidence for the transformation to todorokite, in that the peak at ~2.4 Å begins to split into two peaks at ~2.4 Å and ~2.5 Å, and the peak at ~1.4 Å also beings to show some asymmetry. This agrees with our previous work, in which we transformed Ni-birnessite into todorokite under the same experimental conditions[5]. As the reflux progresses the splitting and asymmetry become more pronounced, there is also a widening of the ~4.8 Å peak, and feint characteristic peaks for todorokite begin to appear at ~2.2, ~1.9, ~1.7 and ~1.5 Å, as expected based on our previous work[5]. To confirm the transformation to todorokite at 4 wk, we subject our final reflux sample to a standard heat treatment, in which the sample is heated to 110°C. At this temperature the ~10 Å interlayers of Mg-phyllomanganate collapse to ~7 Å, while the todorokite tunnel dimensions remain unchanged, thus any Mg-phyllomanganate left in the sample (within the resolution of XRD)

manifests as a change in the ~10 Å peak and the (re)appearance of the ~7 Å peak. Our XRD of the pre and post heat treated 4 wk sample were the same however, thus confirming that todorokite was formed

In this study we performed the same birnessite to todorokite transformation procedure as in our previous work[5], using a Ni-birnessite with similar crystallinity and surface area, and similarly approximately equal proportions of adsorbed and incorporated Ni. Similar crystallinities were confirmed based on XRD, similar surface areas were confirmed based on BET surface area analysis (121 ± 7 m^2/g in this study compared to 102 ± 5 m^2/g in the previous work[5]), and adsorbed vs incorporated Ni was measured using our wet chemical treatment (this work: 57% adsorbed, 43% incorporated, as shown on Fig. 1 main manuscript; our previous work: 54% adsorbed, 46% incorporated[5]). As expected, the birnessite to todorokite transformation XRD patterns and their development therefore follow very similar trajectories, indicating that the transformation process is reproducible. We did not perform transmission electron microscopy (TEM) on the reflux products in this study, but based on the reproducibility of the transformation procedure, we relate our isotope and solution data (shown in Fig. 2 main manuscript) to the stages of transformation that we identified using TEM in our previous work.

During transformation, isotopically heavy Ni is released to solution from the solid phase (Fig. 2 main manuscript). We observe an initial release of isotopically heavy Ni during transformation, heavier by 1.87 ± 0.16‰ (Fig. 2A, B main manuscript), after which the fractionation between solution and mineral decreases to 0.30 ± 0.16‰ (Fig. 2A, B main manuscript), and then after 14 days, while there is no further release of Ni to solution (Fig. C, D main manuscript), $\Delta^{60}Ni_{aqueous\text{-}mineral}$ increases from 0.59 ± 0.16‰ to 0.94 ± 0.16‰ at 28 days (Fig. 2A, B main manuscript). The initial release of isotopically heavy Ni coincides with the onset of todorokite nucleation at ~6 h observed using transmission electron microscopy in our previous work[5], suggesting that isotopically heavy incorporated Ni from the phyllomanganate is released to facilitate kinking of the phyllomanganate layers, necessary to initiate todorokite formation. The presence of incorporated Ni in the phyllomanganate layers inhibits the growth of todorokite due the fact that Ni is not Jahn-teller distorted, thus reducing the kinking, and slowing down the mineral transformation[5].

The following decrease in $\Delta^{60}Ni_{aqueous-mineral}$ is coincidental with a spike in Mn concentration in solution (Fig. 2C main manuscript), and together these coincide with the onset of some phyllomanganate dissolution observed in our previous work[5], suggesting that isotopically light Ni associated with the phyllomanganate is released during the dissolution step. It is during this dissolution step that most of the Ni is released to solution where we observed Ni concentrations of the reflux solution steadily increase from 0.01 mM at 3 h to 0.23 mM after 2 weeks (Fig. 1A. main manuscript) and see a 50% reduction in concentration of the Ni solid (Fig. 1B. main manuscript).

The final increase in $\Delta^{60}Ni_{aqueous-mineral}$ after 14 days and the lack of further release of Ni into solution coincides with the crystal ripening step observed in our previous work[5], during which time any Ni released during dissolution of smaller crystals is likely re-adsorbed onto larger crystals forming. Therefore, the increase in solution $\delta^{60}Ni$ could be due to a kinetic isotope effect during re-adsorption of isotopically light Ni. Using EXAFS spectroscopy our previous work shows that Ni uptake to the neo-todorokite is not via incorporation and is instead via adsorption as inner-sphere complexes located at the edge sites of the todorokite walls, but these complexes are octahedral with no change in the Ni coordination number[5], such that once equilibrium is reached, the $\delta^{60}Ni$ of the solution should stabilise. Overall, at the end of the experiment, we observe an isotopic fractionation between the solution and mineral of $\Delta^{60}Ni_{aqueous-mineral} = +0.9 \pm 0.16‰$.

SUPPLEMENTARY INFORMATION 4

Calculations of the mass balance response to Ni burial in sediments in Figure 4

Figure 4 in the main manuscript is the mass-balance response of seawater Ni isotope composition to mineralogical controls of Ni removal. Figure 4A is a ternary diagram showing the response of seawater isotope compositions as a function of Ni removal by oxic (O), reducing (R), and euxinic (E) sediments. These contour lines are calculated by assuming steady-state, whereby the input and output flux magnitude (equation 1) and their flux weighted isotope composition (equation 2) should be equal. F_{input} and F_{output} are the input and output flux magnitudes respectively and δ_{input} and δ_{output} are their isotopic compositions.

$F_{input} = F_{output}$ (1)

$F_{input}\delta_{input} = F_{output}\delta_{output}$ (2)

In addition, at steady state, the isotopic composition of seawater ($\delta_{seawater}$) can be described as follows:

$$\delta_{\text{seawater}} = \delta_{\text{input}} - \sum f_{\text{output}} \Delta_{\text{output}-\text{seawater}} \qquad (3)$$

In this equation, f_{output} represents the fractional contribution of each sedimentary sink to the total input (i.e., F_{output}/F_{input}), and $\Delta_{output\text{-}seawater}$ is the isotopic offset between the sink and seawater (i.e., $\delta_{output} - \delta_{seawater}$)

The isotopic offset for each sediment sink is as follows: For euxinic sediments, the observed isotopic fractionation between sediments and seawater is consistent with the theoretically calculated fractionation for sulphide precipitation at -0.66‰. Reducing sediments deposited in upwelling regions exhibit the same isotopic composition as modern deep oceans, as such there is no isotopic offset[5,6]. In the modern day, assuming oceanic steady-state and isotopic mass balance, the oxic sediment sink is predicted to have an average δ^{60}Ni of +0.48‰[1], which corresponds to an isotopic fractionation between the oxic sediment sink and seawater ($\Delta^{60}Ni_{oxsed\text{-}sw}$) of -0.85‰. Assuming a modern-day input of 0.8‰ from weathering, using the above values, we can calculate the fraction of Ni removal to each sink for different seawater isotope compositions.

Figure 4B shows a cross plot of seawater δ^{60}Ni values in response to theoretical ranges of Ni isotope fractionation by Mn minerals in oxic sediments. This graph is again calculated using equation 3, assuming modern proportions of Ni removal at 59% to oxic sediments, 38% removal to reducing sediments and 3% removal to euxinic sediments and varying the $\Delta^{60}Ni_{oxsed\text{-}sw}$ value based on the range from Figure 1 in the manuscript.

SUPPLEMENTARY INFORMATION 5

Supplementary Table 4 Ni and Mn concentrations of solid, solution and wash from the reflux experiment.

Sample	Solid			MQ Wash		Solution	
	Mass dissolved (g)	Measured Ni in solution (mg L^{-1})	Ni (wt%)	Measured Ni concentration (µg L^{-1})	Measured Mn concentration (µg L^{-1})	Measured Ni concentration (µg L^{-1})	Measured Mn concentration (µg L^{-1})
Ni-Bn	0.0615	166.60	1.35				
3h	0.0603	169.09	1.19	91	0.09	0.88	876
6h	0.0523	124.72	0.99	346	0.35	2.95	2950
24h[a]	0.057	112.80	0.75	-	-	1.80	1800
48h	0.0786	118.66	0.87	628	0.63	1.65	1650
72h	0.0615	107.13	0.86	882	0.88	0.58	575
1 wk	0.0565	97.03	1.04	1408	1.41	0.49	493
2 wk	0.0543	113.35	0.81	2154	2.15	0.18	179
3 wk	0.0624	101.36	0.84	2792	2.79	0.27	268
4 wk	0.0603	101.38	0.79	1360	1.36	0.88	876

[a] MQ wash measurements for this time point were not obtained due to loss of sample.

Supplementary Table 5 Ni Isotope ratios of the solid, solution and wash from the reflux experiment and mass balance calculations. The starting composition of the solid has a $\delta^{60}Ni$ of 1.11 ‰

Sample	Solid				MQ Wash				Solution				Mass balance[c]
	Total Ni (mg)	F_{Ni}[b]	$\delta^{60}Ni$ (‰)	2σ	Total Ni (mg)	F_{Ni}[b]	$\delta^{60}Ni$ (‰)	2σ	Total Ni (mg)	F_{Ni}[b]	$\delta^{60}Ni$ (‰)	2σ	
Ni-Bn	484	1.000	-1.11	0.04									
3h		0.997	-1.10	0.04	0.02	0.0000	-0.62	0.04	1.42	0.0029	-0.21	0.04	-1.10
6h		0.999	-1.11	0.03	0.07	0.0001	0.01	0.05	0.74	0.0015	0.75	0.06	-1.11
24h[a]		0.996	-1.13	0.04	-	-	-	-	2.02	0.0042	-0.83	0.04	-1.13
48h		0.992	-1.06	0.05	0.13	0.0003	-0.41	0.06	3.66	0.0076	-0.73	0.03	-1.05
72h		0.989	-1.05	0.04	0.18	0.0004	-0.45	0.04	5.30	0.0110	-0.71	0.04	-1.05
1 wk		0.984	-1.10	0.04	0.28	0.0006	-0.45	0.04	7.84	0.0162	-0.58	0.03	-1.09
2 wk		0.978	-1.17	0.04	0.43	0.0009	-0.50	0.03	10.88	0.0225	-0.58	0.03	-1.16
3 wk		0.977	-1.11	0.03	0.56	0.0012	-0.39	0.03	11.20	0.0231	-0.42	0.03	-1.09
4 wk		0.977	-1.15	0.04	0.27	0.0006	-0.26	0.03	11.36	0.0235	-0.21	0.04	-1.13

[a] MQ wash measurements for this time point were not obtained due to loss of sample. Ni mass balance estimates for 24 h exclude the MQ wash.
[b] Solid F_{Ni} is calculated as (Ni-Bn Total Ni – Wash Total Ni –Solution Total Ni)/Ni-Bn Total.
[c] The mass balance is calculated as: $\delta^{60}Ni_{solution} \times f_{solution} + \delta^{60}Ni_{wash} \times f_{wash} + \delta^{60}Ni_{solid} \times f_{solid}$. The $\delta^{60}Ni$ value of the starting Ni-Bn solid is −1.11 ± 0.08‰, based on our long-term reproducibility of our Ni isotope analyses.

References

1. Fleischmann, S. *et al.* The nickel output to abyssal pelagic manganese oxides: A balanced elemental and isotope budget for the oceans. *Earth Planet Sci Lett* 619, 118301 (2023).
2. Little, S. H. *et al.* Towards balancing the oceanic Ni budget. *Earth Planet Sci Lett* 547, 116461 (2020).
3. Knaack, D. R. *et al.* Manganese nodules NOD-A-1 and NOD-P-1: Implications of pre-treatment on oxygen isotopes and mineralogy. *Chem Geol* 558, 119924 (2020).
4. Atkins, A. L., Shaw, S. & Peacock, C. L. Nucleation and growth of todorokite from birnessite: Implications for trace-metal cycling in marine sediments. *Geochim Cosmochim Acta* 144, 109–125 (2014).
5. Atkins, A. L., Shaw, S. & Peacock, C. L. Release of Ni from birnessite during transformation of birnessite to todorokite : Implications for Ni cycling in marine sediments. *Geochim Cosmochim Acta* 189, 158–183 (2016).
6. Silvester, E., Manceau, A. & Drits, V. A. Structure of synthetic monoclinic Na-rich birnessite and hexagonal birnessite: II. Results from chemical studies and EXAFS spectroscopy. *American Mineralogist* 82, 962–978 (1997).
7. Villalobos, M., Toner, B., Bargar, J. & Sposito, G. Characterization of the manganese oxide produced by Pseudomonas putida strain MnB1. *Geochim Cosmochim Acta* 67, 2649–2662 (2003).
